# A role for the JAK-STAT1 pathway in blocking replication of HSV-1 in dendritic cells and macrophages

**DOI:** 10.1186/1743-422X-6-56

**Published:** 2009-05-13

**Authors:** Kevin R Mott, David UnderHill, Steven L Wechsler, Terrence Town, Homayon Ghiasi

**Affiliations:** 1 Center for Neurobiology & Vaccine Development, Ophthalmology Research, Department of Surgery, Cedars-Sinai Medical Center, Los Angeles, CA, USA; 2 Immunobiology Research Institute, Cedars-Sinai Medical Center, Los Angeles, CA, USA; 3 The Gavin Herbert Eye Institute, University of California, Irvine, CA, USA; 4 The Department of Microbiology and Molecular Genetics, University of California, Irvine, School of Medicine, Irvine, CA, USA; 5 Center for Virus Research, University of California, Irvine, USA; 6 Departments of Neurosurgery and Biomedical Sciences, Maxine Dunitz Neurosurgical Institute, Cedars-Sinai Medical Center, Los Angeles, CA, USA; 7 Department of Medicine, David Geffen School of Medicine at UCLA, Los Angeles, CA, USA

## Abstract

**Background:**

Macrophages and dendritic cells (DCs) play key roles in host defense against HSV-1 infection. Although macrophages and DCs can be infected by herpes simplex virus type 1 (HSV-1), both cell types are resistant to HSV-1 replication. The aim of our study was to determine factor (s) that are involved in the resistance of DCs and macrophages to productive HSV-1 infection.

**Results:**

We report here that, in contrast to bone marrow-derived DCs and macrophages from wild type mice, DCs and macrophages isolated from signal transducers and activators of transcription-1 deficient (STAT1^-/-^) mice were susceptible to HSV-1 replication and the production of viral mRNAs and DNA. There were differences in expression of immediate early, early, and late gene transcripts between STAT1^+/+ ^and STAT1^-/- ^infected APCs.

**Conclusion:**

These results suggest for the first time that the JAK-STAT1 pathway is involved in blocking replication of HSV-1 in DCs and macrophages.

## Backgrounds

Macrophages and DCs are bone marrow-derived cells that are involved in antigen capture, processing, and presentation and thus play a key role in triggering the immune system against infectious agents [1-6]. Although both macrophages [7] and DCs [8] cross-present antigens, only DCs are capable of stimulating naive CD8^+ ^T cells [9,10]. DCs also play an important role in initiation of NK anti-viral immunity [11,12]. Similar to DCs, macrophages also play a variety of roles in immune system-mediated defense, including a central role in innate or natural immunity. Macrophages exhibit a wide variety of functions, including phagocytosis, tumor cytotoxicity, cytokine secretion and antigen presentation [13-15]. A number of factors are known that "activate" or engage macrophages in these activities, including viral infection.

Herpes simplex virus (HSV) infections are among the most frequent serious viral infections in the U.S. and are considered to be a major health issue in developed countries [16-19]. Both macrophages and DCs perform crucial roles in linking innate and adaptive immunity and augmenting the immune response to HSV-1 infection. It was previously shown that human blood monocytes are resistant to HSV-1 infection [20-22], although a more recent study reported that immature monocyte-derived human DCs could be moderately infected with HSV-1, resulting in productive infection [23]. Bone marrow-derived macrophages are also resistant to HSV-1 infection [24-28].

The factors involved in the resistance of DCs and macrophages to productive HSV-1 infection are not known. The aim of our study was to determine if STAT1 might play a role in DC and macrophage resistance to HSV-1 replication. We found that DCs and macrophages isolated from STAT1^-/- ^mice lost their resistance to HSV-1 infection. Thus, STAT1 seems to be critically important for allowing DCs and macrophages to resist HSV-1 replication.

## Materials and methods

### Virus, cells, and mice

Triple plaque purified HSV-1 strains McKrae, KOS, and GFP-VP22 were grown in rabbit skin (RS) cell monolayers in minimal essential media (MEM) containing 5% fetal calf serum. GFP-VP22 (a gift from Peter O'hare; Marie Curie Research Institute, Surrey, United Kingdom) is a recombinant virus that contains the gene encoding a major tegument protein, VP22, linked to green fluorescent protein (GFP) [29,30]. Six week old female BALB/c (The Jackson Laboratory), 129SVE-STAT1^-/-^, and 129SVE (Taconic) mice were used as a source of bone marrow (BM) for the generation of mouse DCs and macrophages in cultures. BM cells were isolated by flushing femurs and tibiae with PBS. Pelleted cells were briefly resuspended in water to lyse red blood cells and stabilized by adding complete medium (RPMI 1640, 10% fetal bovine serum, 100 U/ml penicillin, 100 μg/ml streptomycin, 2 mM L-glutamine). The cells were centrifuged and resuspended in complete medium supplemented with either murine Flt3-ligand (100 ng/ml; Peprotech, NJ) or GM-CSF (100 ng/ml; Peprotech, NJ) to enhance replication of DCs [31]. To grow macrophages, the media was supplemented with CSF (100 ng/ml; Peprotech, NJ) instead of Fl3tL or GM-CSF. The cells were plated in non-tissue culture plastic Petri dishes (1 bone per 10 cm dish) for 5 days at 37°C with CO2. After 5 days, the media is removed, the adherent cells were recovered by incubating the cells for 5 min. at 37°C with Versene (Invitrogen, San Diego, CA). Cells were washed, counted, and plated onto tissue-culture dishes for use the following day.

### Virus replication in tissue culture

Monolayers of macrophages or DCs were infected with various amounts of HSV-1 strain McKrae ranging from 0.01 to 10 PFU/cell. One hr after infection at 37°C or 4°C, virus was removed and the infected cells were washed three times with fresh media at the appropriate temperature and fresh media was added to each well. The monolayers including media were harvested at various times by freezing at -80°C. Virus was harvested by two cycles of freeze-thawing and infectious virus titers were determined by standard plaque assays on RS cells as we previously described [32].

### Viral RNA and DNA extraction and cDNA preparation in vitro

DCs or macrophages grown in 24-well plates were infected with 10 PFU/cell of HSV-1 strain McKrae. RNA preparation was done as we previously described [33]. Briefly, frozen cells were resuspended in TRIzol and homogenized, followed by addition of chloroform, and subsequent precipitation using isopropanol. The RNA was then treated with DNase I to degrade any contaminating genomic DNA followed by clean-up using a Qiagen RNeasy column as described in the manufacturer's instructions. The RNA yield from all samples was determined by spectroscopy (NanoDrop ND-1000, NanoDrop Technologies, Inc., Wilmington, Delaware). Finally, 1000 ng of total RNA was reverse-transcribed using random hexamer primers and Murine Leukemia Virus (MuLV) Reverse Transcriptase from the High Capacity cDNA Reverse Transcription Kit (Applied Biosystems, Foster City, CA), in accordance with the manufacturer's recommendations.

DNA isolation was done as we previously described [33]. Briefly, cells from each well in tissue culture media were frozen and thawed 2 times at -80°C prior to processing. The lysed cells from each well were transferred to individual microcentrifuge tubes and centrifuged at 3000 rpm to clear cellular debris. The supernatant was recovered and centrifuged in a microcentrifuge at 14,000 rpm to recover the viral DNA pellet. The pellet was digested for 2 hours at 55°C in TE buffer containing 0.1% SDS and 200 μg of Proteinase K. The mixture was extracted with Phenol/Chloroform followed by subsequent viral DNA precipitation using ethanol.

### TaqMan Real-Time PCR

The expression levels of several viral genes, along with the expression of the cellular GAPDH gene (internal control) were evaluated using commercially available TaqMan Gene Expression Assays (Applied Biosystems, Foster City, CA) with optimized primer and probe concentrations as we previously described [33,34]. Primer-probe sets consisted of two unlabeled PCR primers and the FAM™ dye-labeled TaqMan MGB probe formulated into a single mixture. The HSV-1 ICP0, ICP4, TK, and gB primers and probe used were as follows: 1) ICP0: forward primer, 5'-CGGACACGGAACTGTTCGA-3'; reverse primer, 5'-CGCCCCCGCAACTG-3'; and probe, 5'-FAM-CCCCATCCACGCCCTG-3' – Amplicon length = 111 bp; 2) ICP4: forward primer, 5'-GCGTCGTCGAGGTCGT-3'; reverse primer, 5'-CGCGGAGACGGAGGAG-3'; and probe, 5'-FAM-CACGACCCCGACCACC-3' – Amplicon length = 69 bp; 3) TK: forward primer, 5'-CAGTAGCGTGGGCATTTTCTG-3'; reverse primer, 5'-CCTCGCCGGCAACAAAA-3'; and probe, 5'-FAM-CTCCAGGCGGACTTC-3' – Amplicon length = 59 bp; and 4) gB: forward primer, 5'-AACGCGACGCACATCAAG-3', reverse primer, 5'-CTGGTACGCGATCAGAAAGC-3'; and probe, 5'-FAM-CAGCCGCAGTACTACC-3' – Amplicon length = 72 bp. As an internal control, a set of GAPDH primers from Applied Biosystems (ASSAY I.D. m999999.15_G1 – Amplicon Length = 107 bp) was used.

Quantitative real-time PCR was performed as we described previously [33]. Real-time PCR was performed in triplicate for each sample from each time point. Relative gene expression levels were normalized to the expression of the GAPDH housekeeping gene (endogenous loading control).

### Flow Cytometric Analysis

Infected or mock infected cells were harvested and stained with anti-CD8a-PerCp (clone 53-6.7), anti-CD11b-APC (clone M1/70), anti-CD11c-FITC (clone HL3), anti-CD45R/B220-PerCP (clone RA3-6B2), anti-CD40-PE (clone 1C10), anti-Gr-1-PE (clone RB6-8C5), anti-CD80-FITC (clone 16-10A1), anti-CD83-APC (clone Michel-19), anti-CD86-PE (clone GL1), anti-CD154-PE (clone MR1), anti-MHC class I-FITC (clone 34-1-2S), anti-MHC class II-APC (clone M5/114.15.2), anti-B7-HI-PE (clone MIH5), B7-DC (clone 122), anti-Annexin-PE, and 7-ADD from BD PharMingen (San Diego, CA) and Biolegend (San Diego, CA) and then analyzed by FACS as we previously described [35].

### Confocal Microscopy and Image Analysis

Macrophages or DCs isolated from STAT1-deficient or control 129SVE mice grown on Lab-Tex chamber slides were infected with HSV-1 GFP-VP22 (ranging from 0.01 to 10 PFU for 24 h) as previously reported [29,30]. This GFP-expressing recombinant virus allows for direct monitoring of virus infectivity without additional manipulation. We visualized GFP expression together with F4/80 Ag-PE (as a macrophage marker) or CD11c-PE (as a DC marker) immunostaining 24 h after HSV-1 GFP-VP22 infection. Briefly, cells were fixed by incubating slides in methanol for 10 min followed by acetone for 5 min at -20°C. Afterwards, slides were rinsed three times for 5 min each at ambient temperature in PBS containing 0.05% v/v Tween-20 (PBS-T). Slides were then blocked for 30 min at ambient temperature in PBS-T containing 1% w/v BSA (PBS-TB). Immunostaining was done according to a direct method using F4/80 Ag-PE or CD11c-PE antibodies (1:200 in PBS-TB for 1 h at ambient temperature) (Becton Dickinson). After an additional three rinses at ambient temperature in PBS-T for 5 min each, slides were dipped into ddH_2_O (to remove salt) and mounted in ProLong Gold mounting media containing DAPI (Invitrogen).

Images were captured at 1024 × 1024 pixels (original magnification = 20×) in independent fluorescence channels using a Nikon C1 eclipse inverted confocal microscope. We then exported images (n = 3 per condition) as 8-bit greyscale TIFF files for image analysis using Image J software, release 1.40 g. Quantification of GFP labeling was done by first inverting greyscale images and then using thresholding mode to select positive pixels. Data are represented as % immunolabeled area (positive pixels/total pixels captured × 100%). All analyses were done by a single examiner (T.T.) blinded to sample identities, and code was not broken until the analysis was completed.

### Statistical analysis

Statistics were done by Student's t test or Fisher's exact test using Instat (GraphPad, San Diego, CA). Results were considered to be statistically significant if the p value was < 0.05.

## Results

### HSV-1 replication in DCs isolated from BALB/c mice

**Figure 1:**
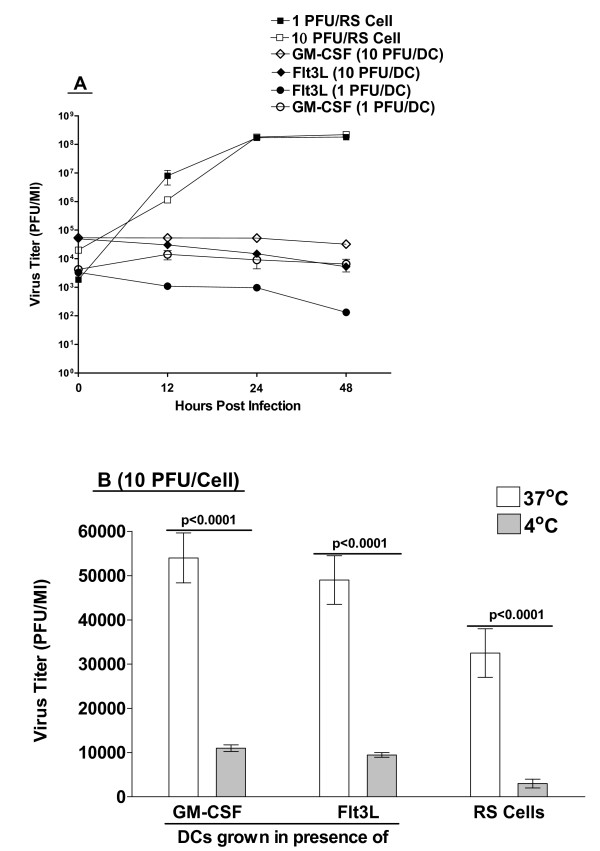
**Replication of HSV-1 in DCs isolated from BALB/cmice**. Panel A. Subconfluent monolayers of DCs and RS cells were infected with 10 or 1 PFU per cell of McKrae and the virus yield determined at the indicated times by standard plaque assays as described in Materials and Methods. Panel B. Cells were infected at 10 PFU per cell and virus allowed to attach for 1 h at 4°C or 37°C. Monolayers were washed 3× and total virus remaining associated with the cells was determined by plaque assay as described in Materials and Methods. In both panels each point represents the mean ± SEM (n = 16) from two to 4 separate experiments.

Previously it was reported that DCs isolated from blood of humans are resistant to HSV-1 infection [20-22]. To determine whether murine bone marrow-derived DCs were also resistant to HSV-1 infection, DCs were isolated from BALB/c mice and cultured in the presence of Flt3L or GM-CSF as described in Materials and Methods. BM-derived DCs are differentially regulated by their growth in Flt3L or GM-CSF [31]. DCs were infected with 1 or 10 PFU/cell of WT HSV-1 strain McKrae. Control RS cells were similarly infected with HSV-1 McKrae. The kinetics of virus replication were quantitated by determining the amount of infectious virus at various times post infection using a plaque assay as described in Materials and Methods. At all MOIs, replication of HSV-1 in DCs was dramatically lower than that seen in RS cells (Fig. 1A). At 48 hrs post-infection, the amount of infectious virus from DC cultures was reduced > 1,000 fold compared to RS cells, suggesting poor virus replication in DCs grown in the presence of Flt3L or GM-CSF. These results were consistent with previous studies showing that human DCs are not permissive to HSV-1 infection [20-22].

### Virus attachment/DC-virus complex formation

To determine if there were possible defects in virus association with DCs, we infected highly permissive RS cells (positive control) and DCs with 10 PFU/cell of McKrae and kept the infected cells at 4°C or 37°C for 1 hr to allow viral attachment. Unbound virus was removed by washing 3× with fresh media at the incubation temperature. The same amount of tissue culture media was added to each cell monolayer in 24 well plates and the cells were frozen at -80°C. After two cycles of freeze-thawing, we determined virus titer by plaque assay. At both 4°C and 37°C, the amount of infectious virus that was detected associated with the DCs was equal to or greater than the amount of infectious virus associated with the RS cells (Fig. 1B). This suggests that the low virus titer in DCs was probably not the result of poor virus attachment to DCs as compared to RS cells.

### Replication of HSV-1 in DCs isolated from STAT1^-/- ^mice

**Figure 2:**
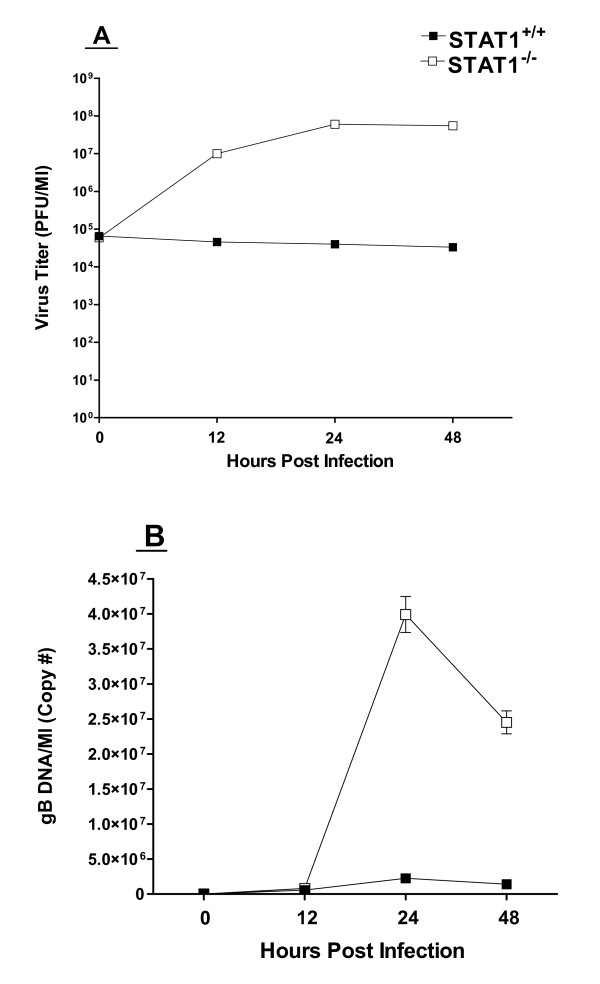
**Replication of HSV-1 in DCs isolated from STAT1^-/- ^mice**. Subconfluent monolayers of DC cells from STAT1^-/- ^and parental Wt STAT1^+/+ ^129SVE mice were infected with 10 PFU/cell as in Fig. 1. Panel A. Virus replication was determined as in Fig. 1. Each point represents the mean ± SEM (n = 16). Panel B. DNA was isolated and the amount of viral genomic DNA was determined by Taq-Man PCR as described in Materials and Methods and normalized to GAPDH DNA. Each point represents the mean ± SEM (n = 6). Note that the DNA levels are normalized to the levels present one hour after virus is first added to the cell monolayer (the adsorption period), a time is routinely taken as t = 0. However, significant levels of ICP0 and ICP4 DNA are already present at this time (Ct of 20–21) which masks these DNA levels at early times.

Our results with BALB/c mice described above suggested that DCs isolated from wt BALB/c mice were not permissive to HSV-1 infection. To determine if BM-derived DCs from STAT1^-/- ^mice were susceptible to HSV-1 infection, we isolated DCs from STAT1^-/- ^mice (129SVE background), cultured them in the presence of GM-CSF, and infected them with 10 PFU/cell of HSV-1 McKrae as described above. We included DCs isolated from parental STAT^+/+ ^mice (wild-type 129SVE mice) as controls. Virus replication in the STAT1^-/- ^DCs (Fig. 2A; open squares) was approximately 1,000-fold higher at 48 hrs post-infection than in DCs from 129SVE mice (solid squares; Fig. 2A). Virus replication in DCs from wild type 129SVE mice was similar to that seen in DCs from wild type BALB/c mice (compare solid squares in Fig. 2A to open diamonds in Fig. 1A). In addition, HSV-1 replication in the STAT1^-/- ^DCs approached that seen in RS cells. In our experience, RS cells support replication of wild type HSV-1 McKrae as well or better than any other cell line. Thus, STAT1^-/- ^DCs appear to support HSV-1 replication with high efficiency.

### Viral DNA in STAT1^-/- ^compared to normal DCs

To further confirm the titration results described above (Fig. 2A), we also determined the amount of gB DNA as a measure of the relative amount of viral genomic DNA (Fig. 2B). Infected STAT1^-/- ^DCs had significantly more gB DNA than the STAT^+/+ ^parental 129SVE DCs (p < 0.05). This was also consistent with reduced HSV-1 replication in wild type DCs compared to STAT1^-/- ^DCs, and suggests that the block in HSV-1 replication in wild type DCs occurs prior to viral DNA replication.

### Viral transcription in STAT1^-/- ^compared to normal DCs

**Figure 3:**
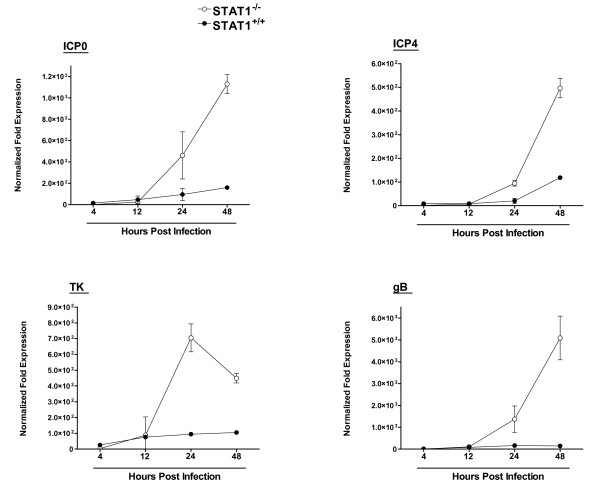
**Level of HSV-1 immediate early, early, and late viral transcripts in DCs isolated from STAT1^-/- ^mice**. Subconfluent monolayers of DC cells from STAT1^-/- ^and parental Wt STAT1^+/+ ^129SVE mice were infected with 10 PFU/cell as in Fig. 1. Total RNA was isolated and TaqMan RT-PCR was performed using ICP0-, ICP4-, TK-, and gB-specific primers as described in Materials and Methods. ICP0, ICP4, TK, and gB mRNA levels were normalized in comparison to each transcript at 0 hr post infection. GAPDH was used as internal control. Each point represents the mean ± SEM (n = 16) from three separate experiments for gB and two experiments for ICP0, ICP4, and TK. Note that the mRNA levels are normalized to the levels present one hour after virus is first added to the cell monolayer (the adsorption period), a time is routinely taken as t = 0. However, significant levels of ICP0 and ICP4 mRNA are already present at this time (Ct of 20–21) which masks these mRNA levels at early times.

The above results suggest that, while DCs from both STAT1^-/- ^and STAT^+/+ ^mice are permissive to infection with HSV-1, the virus only replicates efficiently in STAT1^-/- ^DCs. Since HSV-1 replicates in a temporal cascade of three classes of viral genes: immediate-early (IE), early (E) and late (L) genes, we looked at the possibility of blockage of transcription of one or more of these three classes of viral genes in DCs isolated from STAT^+/+ ^mice. We infected STAT1^-/- ^and STAT^+/+ ^DCs with 10 PFU/cell of HSV-1 McKrae and then harvested cells at 0, 4, 12, 24, and 48 hr post-infection. Total RNA was isolated as described in Materials and Methods, and various viral mRNA levels were quantitated by RT-PCR. ICP0 and ICP4 were used as indicators of IE genes, TK as an example of an E gene, and gB was taken as a late gene. We performed TaqMan RT-PCR on isolated RNA to determine the amount of HSV-1 ICP0, ICP4, TK, and gB mRNAs relative to levels of each transcript at baseline (just prior to infection). Cellular GAPDH mRNA was used as an internal control. Our results suggest that between 4 hr and 12 hr PI the levels of ICP0 (Fig. 3, ICP0), ICP4 (Fig. 3, ICP4), TK (Fig. 3, TK), and gB (Fig. 3, gB) transcripts were similar between STAT1^-/- ^and STAT1^+/+ ^DCs. However, by 24 and 48 hrs PI the levels of ICP0 (Fig. 3, ICP0), ICP4 (Fig. 3, ICP4), TK (Fig. 3, TK), and gB (Fig. 3, gB) transcripts in STAT1^-/- ^DCs were significantly higher than that seen in STAT1^+/+ ^DCs (p < 0.05). These results were consistent with increased viral replication in the STAT1^-/- ^DCs compared to wild-type DCs, and suggest that the block to virus replication in wild-type DCs occurs between 12 hr and 24 hr PI. Overall, the results for viral replication, viral DNA, and viral mRNA, are all consistent with STAT1 being involved in the resistance of normal DCs to HSV-1 replication.

### Effect of HSV-1 infection on cell surface markers on wild type and STAT1^-/- ^DCs

**Figure 4:**
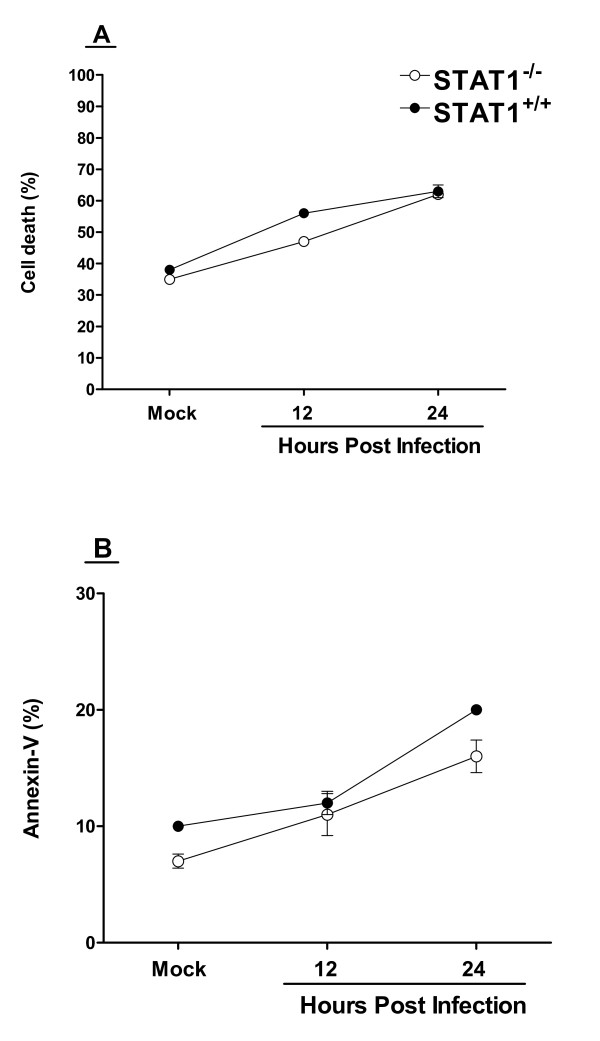
**FACS analyses of isolated DCs**. Subconfluent monolayers of DCs isolated from STAT^-/- ^and parental STAT^+/+ ^129SVE mice grown in GM-CSF containing media were infected with 10 PFU/cell of McKrae. At the indicated times post infection the cells were harvested and reacted with Annexin-V or 7-ADD dye to analyze apoptosis and cell death respectively and FACS analysis was performed (see Materials and Methods). Since DCs isolated from STAT^-/- ^mice did not survive to 48 h post infection, FACS was done at 12 and 24 hr post infection for STAT1^-/- ^parental STAT^+/+ ^129SVE DCs. The percent of cells positive are shown. The results are the average of two experiments.

To investigate potential differences in DC maturation in STAT1^-/- ^compared to STAT^+/+ ^cells, we isolated DCs from STAT1^-/- ^and STAT^+/+ ^mice, infected them with 10 PFU/cell of HSV-1 strain McKrae, and assessed cell-surface markers by flow cytometry. The percent of DCs staining for the cell death marker propidium iodide (Fig. 4A) and the apoptosis marker Annexin V (Fig. 4B) appeared similar in the parental 129SVE (STAT1^+/+^) DCs and STAT1^-/- ^DCs. Similarly, there was no evidence of increased staining for any other markers tested, including CD11b, CD45R/B220, CD40, Gr-1, CD80, CD83, CD86, CD154, MHC class I, MHC class II, B7-HI, B7-DC, and CD8α in the STAT1^-/- ^DCs compared with DCs isolated from wild-type mice (data not shown). Overall, we did not find evidence of association between specific cell surface marker(s) and susceptibility of STAT1^-/- ^DCs to HSV-1 infection.

### HSV-1 replication in BM-derived macrophages isolated from BALB/c mice

**Figure 5:**
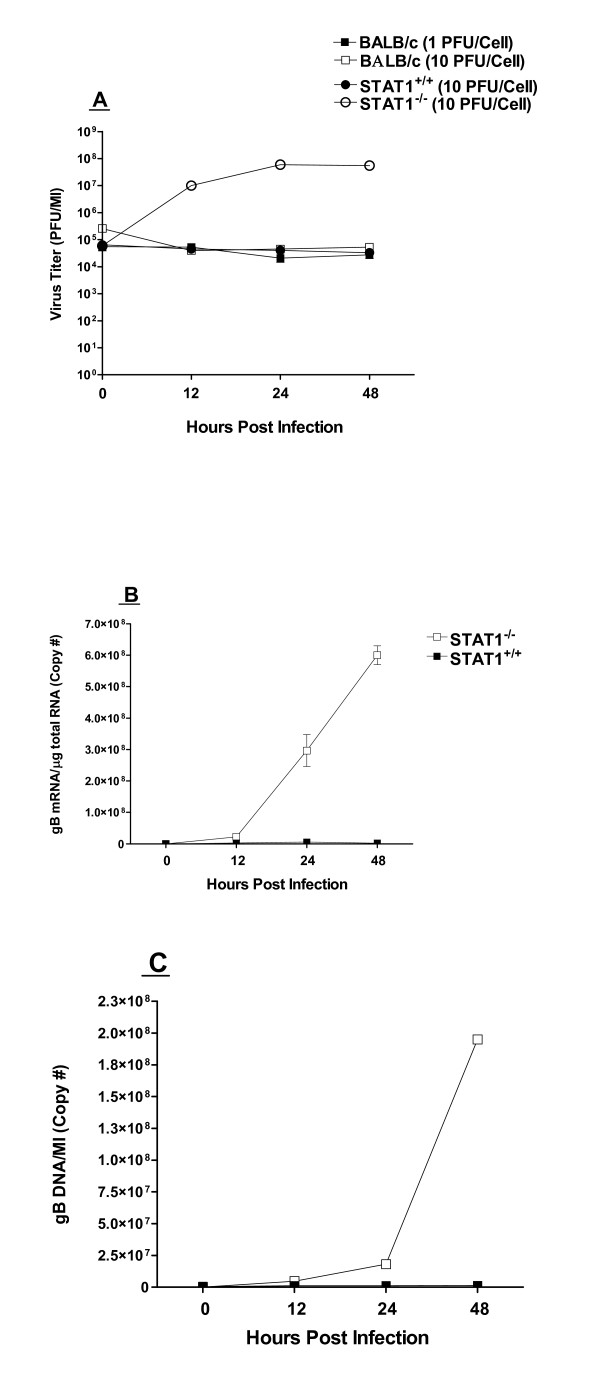
**Replication of HSV-1 in macrophages**. Analyses of virus replication, viral gB mRNA, and viral genomic DNA in macrophages was done as in Fig. 2 for DCs. Panel A. Virus replication, n = 12. Panel B. gB mRNA, n = 6. Panel C. Viral genomic DNA, n = 6. The results are the average of two experiments.

Similar to DCs, BM-derived macrophages have also been reported to be nonpermissive to HSV-1 infection [24-28]. To determine if BM-derived macrophages from BALB/c mice were also resistant to HSV-1, macrophages were cultured as described in Materials and Methods and infected with HSV-1 strain McKrae. The yield of infectious virus was quantitated as above. We did not detect significant virus replication at any infectious dose (Fig. 5A, 1 or 10 PFU/cell; 0.1 and 0.01 PFU/cell, not shown). Thus, similar to BALB/c DCs, BALB/c macrophages did not appear permissive to HSV-1 infection.

### Macrophages isolated from STAT1^-/- ^mice are susceptible to HSV-1 infection

Macrophages were isolated from STAT1^-/- ^mice and parental 129SVE mice and infected with 10 PFU/cell of HSV-1 strain McKrae. Similar to the results described above for DCs, macrophages from STAT1^-/- ^mice were more susceptible to HSV-1 infection than macrophages from wild-type 129SVE mice as judged by virus yield (Fig. 5A), levels of gB mRNA (Fig. 5B), and the amount of genomic DNA (Fig. 5C). As with DCs from STAT1^-/- ^mice, virus replication in macrophages from these mice approached that seen in highly-susceptible RS cells. Thus, in the absence of STAT1, HSV-1 replication in murine macrophages and DCs was highly efficient.

### Detection of GFP expression in infected DCs and macrophages by confocal microscopy

**Figure 6:**
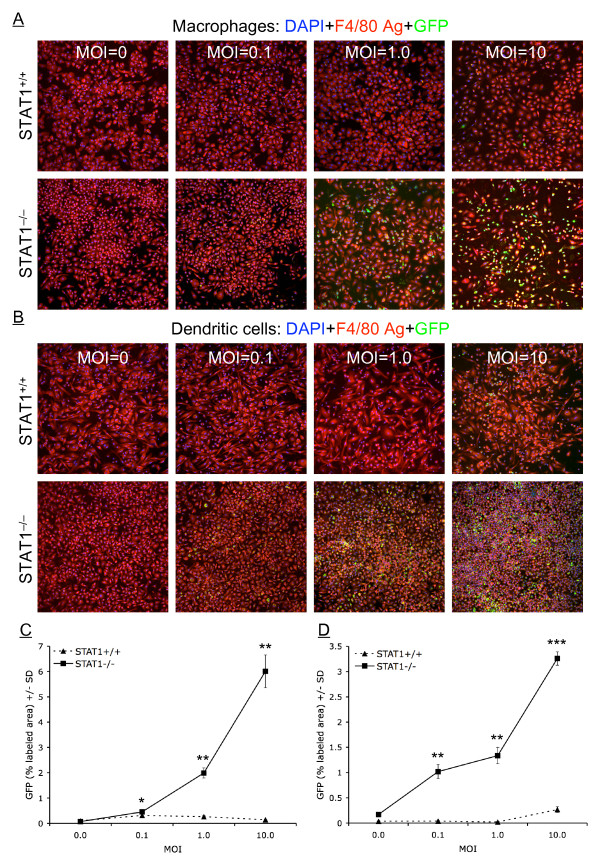
**Confocal analysis of APCs infected with a recombinant HSV-1 expressing GFP**. DCs and macrophages isolated from STAT1^-/- ^or STAT1^+/+ ^wild-type control mice were infected with GFP-VP22 virus. Twenty-four hours post infection, cells were reacted with anti-CD11c-PE or anti-F40/80 Ag-PE mAb. Panel A. Macrophages stained with F4/80 Ag-PE (red signal) and nuclear counter-stained with DAPI (blue signal), revealing GFP (green signal). Panel B. DCs stained with CD11c-PE (red signal) and nuclear counter-stained with DAPI (blue signal), revealing GFP (green signal). Quantification of GFP signal (% labeled area, means ± SD, n = 3 images per condition) is shown for macrophages in Panel C and DCs in Panel D. Similar results were observed in independent experiments.

To further confirm that APCs isolated from STAT1^-/- ^mice are permissive to HSV-1 replication, we infected monolayers of DCs or macrophages isolated from STAT^-/- ^and wild-type control 129SVE mice with 0.1, 1.0 or 10 PFU of GFP-VP22 virus for 24 hr as described in Materials and Methods. We used mAbs against DCs (anti-CD11c-PE mAb) and macrophages (anti-F4/80 Ag-PE mAb) to show specificity of HSV-1 infected GFP-positive DCs and macrophages, respectively. When considering either macrophages (Fig. 6A) or DCs (Fig. 6B), confocal images showed a qualitative increase in GFP labeling at each dose of HSV-1 GFP-VP22. Furthermore, infected cells appeared morphologically distinct (often displaying a more round, activated phenotype) from non-infected cells when considering either genotype. Finally, quantitative image analysis revealed statistically significant increased GFP labeling in STAT1^-/- ^macrophages (by as much as ~40-fold at MOI = 10, Fig. 6C) and in STAT1^-/- ^DCs (by as much as ~60-fold at MOI = 1.0, Fig. 6D) across all three doses of virus administered. Thus, confocal microscopy confirmed our results for viral replication, viral DNA, and viral mRNA, suggesting that STAT1 is involved in the resistance of normal DCs to HSV-1 replication.

## Discussion

It was previously reported that freshly isolated peripheral blood monocytes and lymphocytes are resistant to HSV infection [20-22]. Similarly, infection of resident peritoneal macrophages with HSV-1 results in an abortive infection in which the viral DNA is not replicated and no infectious virus is produced [24-28]. Another study showed that while both mature and immature monocyte-derived DCs are infected by HSV-1, only immature DCs produce infectious virus, but at ten-fold lower levels than most cell lines, despite the fact that DCs express HSV receptors [36]. However, the mechanism of DC and macrophage resistance to HSV-1 replication is not known. Mature DCs are also resistant to productive infection with influenza virus [37] and dengue virus [38]. We show here that BM-derived DCs and macrophages isolated from wild type mice, including BALB/c or 129SVE strains and C57BL/6 strain (data not shown) do not support replication of HSV-1 as judged by virus yield, viral mRNA transcription, confocal microscopy of a GFP-viral fusion protein, and viral genomic DNA levels. However, in DCs from STAT1^-/- ^mice, HSV-1 replication approached that seen in RS cells. We report here that HSV-1 attaches as efficiently to DCs as it does to the highly permissive RS cells, suggesting that the DCs non-permissiveness for HSV-1 is not due to a defect in viral attachment. Thus, non-permissiveness of DCs to HSV-1 appears due to either inefficient virus penetration or a block in the virus' replication cycle. Since it seems more likely that STAT1 would affect virus replication, we lean towards this explanation. However, it should be noted that the experiments reported here do not definitively distinguish between a block in virus entry versus a block in virus replication. Since RS cells support very efficient replication of HSV-1, these results suggest that STAT1 plays a key role in the resistance of DCs and macrophages to HSV-1 replication. We would predict that STAT1 may also be involved in resistance of DCs to influenza virus and dengue virus, since *Chlamydia trachomatis *also propagates more efficiently in STAT1-null or STAT1 knockdown cells [39].

STAT1-deficient mice are highly sensitive to infection by microbial pathogens and viruses [40-44] including HSV-1, which replicates to approximately 1000-fold higher titers in the eyes of STAT1^-/- ^mice compared to wt mice [45]. We obtained similar results when STAT1^-/- ^mice were ocularly infected with HSV-1 strain McKrae or KOS (data not shown). The experiments presented here constitute the first report of a virus replicating more efficiently in DCs and macrophages from STAT1 deficient mice. In addition, the increased HSV-1 replication in DCs and macrophages from STAT1^-/- ^mice was approximately 1000-fold higher than in wild type mice, similar to that reported in the eyes of STAT1^-/- ^mice [45,46]. This raises the possibility that the enhanced sensitivity of STAT1 deficient mice to viruses and particularly the 1000 fold increase in HSV-1 replication in STAT1 deficient mice may be due, at least in part, to increased replication of virus in DCs and macrophages. In this regard, it has been reported that HSV-infected DCs are compromised by the infection process and have reduced T-cell stimulatory capacity [47-49].

The increased replication of HSV-1 in STAT1^-/- ^DCs and macrophages shown here might be an important factor contributing to increased susceptibility of STAT1^-/- ^mice to infection. However, since transfer of BM-derived DCs or macrophages from wild-type mice to STAT1-deficient mice did not reduce the susceptibility of STAT1-deficient mice to HSV-1 infection, even when the avirulent HSV-1 strain KOS was used for ocular challenge (data not shown), additional factors are likely involved. Surprisingly, STAT1^-/- ^and STAT1^+/+ ^mice had similar levels of corneal scarring following ocular HSV-1 infection (data not shown). Thus, the resistance of APCs in STAT1^+/+ ^mice to HSV-1 replication compared to the permissiveness of APCs in STAT1^-/- ^mice to HSV-1 replication, did not appear to play an important role in protecting mice against either death or corneal scarring.

STAT1 is one of the seven members of the mammalian STAT family. STATs participate in gene control and are activated when cells encounter various extracellular polypeptides [50]. Targeted disruption of the STAT1 gene in mice revealed a role for STAT1 in the JAK-STAT signaling pathway [40]. The JAK-STAT signaling pathway is involved in mediating biologic responses induced by many cytokines [51]. STAT1-deficient mice lack responsiveness to IFN-α and IFN-γ [40-44]. Thus, it is possible that the absence of responsiveness to IFN-α or IFN-γ in DCs and macrophages isolated from STAT1-deficient mice contributes to their susceptibility to HSV-1 infection. However, IFN-α production does not appear to correlate with innate protection against HSV-2 [52] and we previously showed that expression of murine IFN-γ by an HSV-1 recombinant virus does not impair virus replication [53,54]. Furthermore, incubation of STAT1^+/+ ^DCs or macrophages in the presence of anti-IFN-α mAb, anti-IFN-γ mAb, or both mAbs did not increase HSV-1 replication in infected DCs (data not shown).

## Conclusion

We have shown here for the first time that STAT1 (likely via the JAK-STAT1 pathway) is involved in suppressing HSV-1 replication in murine DCs and macrophages. In addition, the lack of virus replication in wild-type APCs did not appear to be due to transcriptional blockage of HSV-1 α, β, or γ genes, although viral DNA replication did not occur.

## Competing interests

The authors declare that they have no competing interests.

## Authors' contributions

KM was responsible for conducting experiments. DU was responsible for experimental design and conducting experiments. SW was responsible for writing the manuscript. TT was responsible for experimental design and conducting experiments. HG was responsible for experimental design and writing the manuscript
